# A case report of undiagnosed postpartum hemolytic uremic syndrome

**DOI:** 10.1186/s13000-015-0278-0

**Published:** 2015-07-08

**Authors:** Jiao Mu, Ji Zhang, Ananda Sunnassee, Hongmei Dong

**Affiliations:** Department of Forensic Medicine, Tongji Medical College of Huazhong University of Science and Technology, No. 13 Hangkong Road, Wuhan, Hubei 430030 PR China; Department of Phatology, Hebei North University, No. 11 Zuanshinan Road, Zhangjiakou, Hebei 075000 PR China; Ministry of Health and Quality of Life, Victoria Hospital, Quatre Bornes, Mauritius

**Keywords:** Thrombotic microangiopathy, Hemolytic uremic syndrome, Postpartum hemolytic uremic syndrome, Acute renal failure, Hemolytic anemia

## Abstract

**Background:**

Postpartum hemolytic uremic syndrome (PHUS) is a severe thrombotic microangiopathy (TMA) that is clinically characterized by hemolytic anemia, renal dysfunction, and low platelet levels after childbirth. Here, we report a rare case of unexpected death due to PHUS.

**Case Presentation:**

A 23-year-old parturient had an uncomplicated cesarean section at 40 weeks gestation. The immediate postpartum course was uneventful. However, eight days post delivery, the patient developed severe nausea and vomiting followed by hematuria, spontaneous bruising, marked pallor, icteric sclera, and lethargy. Laboratory findings revealed that the patient had hemolytic anemia, thrombocytopenia, and acute renal failure. This patient died approximately 29 h after the onset of symptoms. Post-mortem examination confirmed that the patient had PHUS.

**Conclusions:**

This paper addresses the need for a renal histological examination in addition to a thorough clinical history and appropriate laboratory tests for the rapid and accurate diagnosis of PHUS. Early detection and diagnosis can significantly improve the prognosis and optimize maternal outcomes.

## Background

The hemolytic uremic syndrome (HUS), first described in 1955 by Gasser, is characterized by microangiopathic hemolytic anemia, thrombocytopenia, and acute renal failure [1]. Pregnancy may be a risk factor for acute episodes of HUS because pregnancy is associated with increased procoagulant factors, decreased fibrinolytic activity, loss of endothelial cell thrombomodulin, and decreased activity of ADAMTS13 [2]. Robson first described this event as postpartum hemolytic uremic syndrome (PHUS) in 1968 [3]. Currently, the pathogenesis of PHUS is not completely known. Although PHUS is better recognized today than in the past years, it is still a life-threatening illness if not treated in time. The occurrence of PHUS is estimated to be 1 per 25,000 pregnancies [4]. Herein, we report the case of a puerpera who developed PHUS following an uncomplicated cesarean section. To the best of our knowledge, very few autopsy-confirmed cases of maternal death due to PHUS have been reported. The postmortem findings, pathology, and potential difficulties in the diagnosis of PHUS have been discussed in the present paper.

## Case presentation

### Case history

A 23-year-old woman, gravida 1, para 0, had an uncomplicated cesarean section at 40 weeks gestation at a county hospital. Her prenatal and medical histories were unremarkable and the immediate postpartum course was uneventful. Eight days after delivery, the patient complained of severe nausea and vomiting. Her vital signs were normal, including a temperature of 36.5°C and blood pressure of 130/80 mmHg. Laboratory examinations revealed a white blood cell (WBC) count of 14.8 × 10^9^/L, platelet (PLT) count of 60 × 10^9^/L, and hemoglobin (Hgb) level of 75 g/L. The patient was given a tentative diagnosis of acute gastroenteritis and mild anemia. However, over the next ten hours, the patient showed an acute deterioration, accompanied by slight vaginal bleeding, gross hematuria, spontaneous bruising, marked pallor, icteric sclera, drowsiness, and even a loss of consciousness. Laboratory tests documented a Hgb level of 60 g/L, RBC of 1.86 × 10^12^/L, PLT of 31× 10^9^/ L, blood urea nitrogen (BUN) of 10.8 mmol/L (normal range: 1.7-8.3 U/L), and total bilirubin (TBIL) of 39.2 umol/L (normal range: 2–23 umol/L). Urinalysis revealed blood [3+] and protein [2+]. Coagulation studies, C-reactive protein (CRP), and serum electrolytes were within the normal range. Craniocerebral computed tomography (CT) examination was also normal. Direct and indirect Coombs tests were both negative.

Given the rapid progression and lack of a definitive diagnosis, the patient was transferred to a provincial hospital. The patient’s blood pressure was 140/90 mmHg. Subsequent investigations revealed hemolytic anemia, with Hgb of 55 g/L and serum lactic dehydrogenase (LDH) of 5286 U/L (normal range 135–214 U/L). There was a rapid deterioration in renal function: creatinine kinase (CK) 349 U/L, creatinine 108 umol/L, urea 13.57 mmol/L, and uric acid 466.7 umol/L. PLT were markedly reduced (5.2 × 10^9^/L) with an increased WBC count of 32.41 × 10^9^/L and an elevated glutamic oxaloacetic transaminase (AST) of 149 U/L. Activated partial thromboplastin time (APTT), thrombin time (TT), prothrombin time (PT), fibrinogen (FIB), and antithrombin (AT) were within the normal range.

Despite aggressive resuscitation attempts over the course of two hours, the patient died. The presumptive diagnoses included disseminated intravascular coagulation (DIC), acute fatty liver of pregnancy (AFLP), hemolysis, elevated liver enzymes, low platelets (HELLP syndrome), and thrombotic thrombocytopenic purpura (TTP). The antemortem peripheral blood smear showed fragmented red blood cells (RBC).

### Autopsy findings

**Figure 1:**
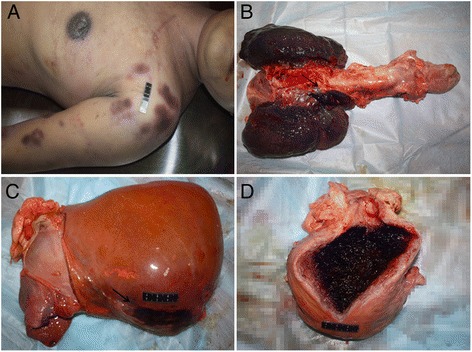
Autopsy findings. **A**: multiple petechial hemorrhages around the shoulder and upper extremity; **B**: focal hemorrhage in the soft tissue around the left lung hilum; **C**: focal hemorrhage in the right lobe of the liver; **D**: hematometra in the uterine cavity.

The body remained frozen (−20°C) until a forensic autopsy was performed two months postmortem because of a medical dispute and an issue of autopsy consent by the family. External examination revealed multiple petechial hemorrhages around the shoulder and extremities. Internal examination was significant for the presence of petechial hemorrhage involving the pericardium, the soft tissue around the left lung hilum, the right lobe of the liver and the uterine cavity (Figure 1). Other findings included 100 mL of serous fluid in the pericardial cavity and 400 mL of dark red fluid in both pleural cavities.

### Histological and toxicological examination

**Figure 2:**
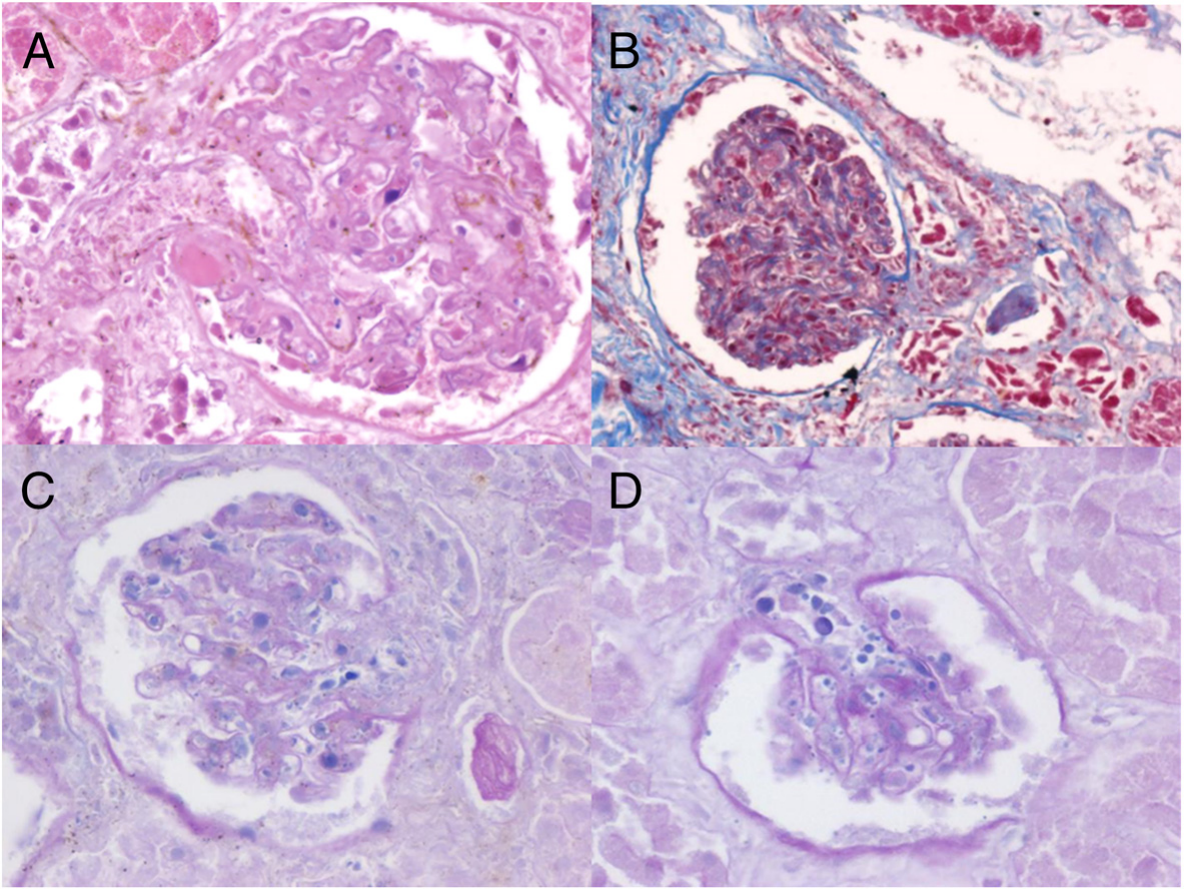
Histological findings of the kidney. **A**, **B**, **C**: multiple fibrin thrombi in glomerular capillaries and afferent arterioles of glomerulus were observed. Focal segmental mesangial proliferation and thickening of capillary walls were also seen. (**A**, H&E stain, ×200; **B**, Masson stain, ×400; **C**, PAS stain, ×400); **D**: collapse of the capillary loops and glomerular ischemia and shrinkage (PAS stain, ×400).

**Figure 3:**
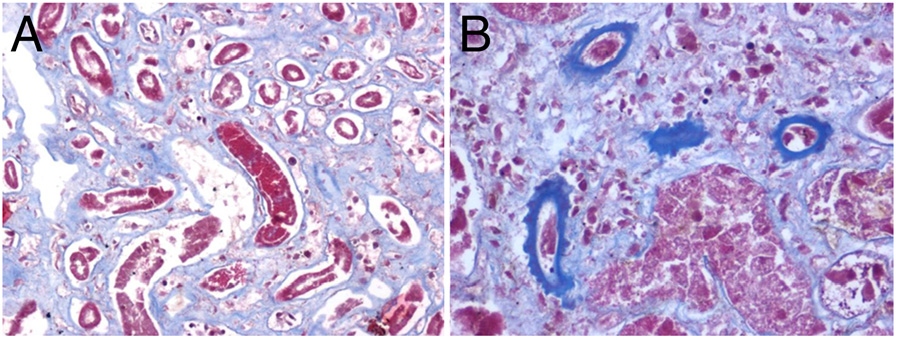
Histological findings of the kidney. **A**: formation of casts in the renal tubule (Masson stain, ×200); **B**: renal arteriolar expansion with fibrin exudation on the arteriolar wall (Masson stain, ×400).

On microscopic examination, both kidneys showed autolysis and diffused glomerular changes with thickening of the capillary walls. There was an increase in the mesangial cell matrix, and segmental obstruction of glomerular capillaries and the renal afferent arteriole by the microthrombus. A number of glomeruli exhibited ischemic changes and atrophy (Figure 2). Protein casts were present in the renal tubules. The fibrin exudation on the renal arteriolar wall was observed (Figure 3). Other organs were unremarkable on gross or microscopic examination. The toxicology analysis revealed no positive findings.

Based on the clinical history, laboratory testing, and autopsy findings, we concluded that the cause of death was PHUS.

## Discussion

PHUS usually occurs in primigravida with a mean age of 27.0 ± 6 years [5]. Patients with PHUS usually experience an uneventful pregnancy and parturition, but typically develop ecchymoses, scleral icterus, hematuria, and anemia after a symptom-free interval [6]. PHUS is a disease that has a poor prognosis and a rapid progression; the median time from onset to death is about 8.5 days [7]. Renal pathology usually shows thickened glomerular capillary walls and expanded renal arteriolar intima with fibrin exudation on the arteriolar wall [6]. In the present study, the age, time of onset, and clinical manifestations were consistent with the literature. Combined with our autopsy findings (petechial hemorrhages of skin and viscera) and renal histological examination, the cause of death was determined to be PHUS.

The diagnosis of PHUS is difficult due to complicated clinical manifestations, which results in misdiagnosis rates as high as 80% following the initial work-up [8]. Consequently, appropriate treatment is usually delayed, leading to a high maternal mortality rate. In the current case, the patient had an insidious onset and rapid progression, with the disease course lasting 29 h from onset to death. Finally, the patient was misdiagnosed with AFLP, HELLP, DIC, and TTP, which are the differential diagnoses for the present clinical symptoms.

Histopathological findings can exclude the possibility of DIC and AFLP. DIC is mostly associated with the primary diseases, such as infection and tumor. The microthrombi are often formed within small vessels in DIC. In the present case, the source of the infection and tumor was not found and the CRP levels were within the normal range. Microthrombi within small vessels of multiple organs were not observed, which ruled out DIC. AFLP is characterized by nausea, vomiting, jaundice, abdominal pain, ecchymosis, central nervous system dysfunction, and renal failure. Histologically, the liver shows fatty metamorphosis [9]. Although the patient in this case report had similar clinical symptoms to those observed in patients with AFLP, the absence of steatosis of the liver excluded a diagnosis of AFLP.

HELLP is characterized by hemolysis, elevated liver enzymes, and thrombocytopenia, and the abnormal biochemical indices are very similar to those of PHUS [10]. However, HELLP is more common in multiparous women and approximately 70% of such cases occur prior to term [11]. Moreover, patients with HELLP frequently present with severe right upper quadrant pain; and liver is the primarily affected organ. Based on these characteristics, combined with the patient’s medical history and postmortem findings, HELLP syndrome was considered to be very unlikely, although a mild increase in transaminases was observed.

TTP is a rare hematologic disorder characterized by microangiopathic hemolytic anemia, thrombocytopenia, neurologic manifestation, fever, and renal disease. Since TTP and PHUS share many clinical features, it is difficult to separate them into two distinct entities. However, TTP usually occurs in the first trimester, while PHUS is typically seen in the postpartum period. Unlike TTP, PHUS has relatively fewer neurologic deficits, but usually has severe renal involvement. Furthermore, the organs affected by the microthrombi are widespread in TTP patients, but are primarily confined to the kidneys in PHUS patients [12]. In this case, the patient developed TMA with disturbance of consciousness that seemed to suggest the presence of TTP. However, the prominent renal insufficiency and the lack of diffused thrombi in multiple organs supported a diagnosis of PHUS rather than TTP.

PHUS is associated with high mortality. Nevertheless, with early diagnosis and appropriate treatment, maternal mortality may be reduced by as much as 90% [13]. In our case, the unexpectedness, suddenness, fulminant course, as well as absence of remarkable clinical manifestations confused the physicians and led to wrong diagnoses and delayed correct treatment. Histopathological finding of endothelial cell injury in renal micro vessels is characteristic features of PHUS and is considered as its definite diagnostic criteria. The current case highlights the importance of raising awareness amongst medical staff, especially obstetricians, to ensure that PHUS is recognized as early as possible. To formulate a correct diagnosis among the possible causes, renal biopsy is suggested. Speed and accuracy of diagnosis are crucial to optimize maternal outcomes.

## Conclusion

In conclusion, the report is a very rare case of a puerpera who developed PHUS following an uncomplicated cesarean section. This paper addresses the need for a renal histological examination in addition to a thorough clinical history and appropriate laboratory tests to diagnose PHUS quickly and accurately.

## Consent

The informed consent was obtained from the patient’s relatives for the publication of this case report.

## Abbreviations

PHUS: Postpartum hemolytic uremic syndrome
TMA: Thrombotic microangiopathy
WBC: White blood cell
PLT: Platelet
Hgb: Hemoglobin
BUN: Blood urea nitrogen
TBIL: Total bilirubin
CRP: C-reactive protein
CT: Computed tomography
LDH: Lactic dehydrogenase
CK: Creatinine kinase
AST: Glutamic oxaloacetic transaminase
APTT: Activated partial thromboplastin time
TT: Thrombin time
PT: Prothrombin time
FIB: Fibrinogen
AT: Antithrombin
DIC: Disseminated intravascular coagulation
AFLP: Acute fatty liver of pregnancy
HELLP: Hemolysis, elevated liver enzymes, low platelets
TTP: Thrombotic thrombocytopenic purpura
